# ForestSeg3D: A semantically guided framework for individual tree segmentation from LiDAR point clouds

**DOI:** 10.1016/j.plaphe.2026.100261

**Published:** 2026-07-23

**Authors:** Ke Zhang, Wenjun Zhang, Weipeng Jing, Liang Li, Chao Li

**Affiliations:** a School of Computer Science and Artificial Intelligence, Northeast Forestry University, Harbin, 150040, China; b China Unicom Smart City Research Institute, Beijing, 100102, China

**Keywords:** Individual tree segmentation, LiDAR point cloud, Semantic segmentation, Knowledge distillation, Forest inventory

## Abstract

Individual tree segmentation from LiDAR point clouds is critical for forest inventory and ecological monitoring. However, accurate delineation remains challenging in complex forest environments with dense crown overlap, occlusion, and multilayer vertical structures. To address these challenges, we propose ForestSeg3D, a semantically guided framework that improves individual tree segmentation through hierarchical semantic supervision and bidirectional cross-task distillation. Specifically, hierarchical semantic supervision (HSS) introduces a coarse-to-fine semantic learning scheme with explicit cross-level consistency, where coarse supervision distinguishes Tree from Non-Tree and fine-grained supervision further decomposes scenes into Ground, Wood, and Leaf, providing structured semantic priors for instance learning and enabling more discriminative tree representations. Building on this representation, bidirectional cross-task distillation (BCTD) explicitly couples semantic prediction and instance partition, reducing conflicts between semantic labels and instance boundaries and improving delineation in crowded forest scenes. To support large-scale inference, ForestSeg3D further incorporates semantic-aware region merging (SRM), a scene-level consolidation strategy that alleviates cross-region conflicts caused by overlapping crowns and boundary-crossing trees. Extensive experiments on the FOR-instanceV2 dataset show that ForestSeg3D achieves the best overall performance in detection completeness, false-positive suppression, and F1-score, while maintaining competitive mAP. The method also maintains strong performance on the ForestSemantic dataset under three-fold leave-one-plot-out cross-validation. Improved segmentation further enables more reliable estimation of tree-level structural attributes, including tree height, crown diameter, and crown volume. These results demonstrate the effectiveness and practical value of ForestSeg3D for individual tree segmentation and downstream forest structural assessment from LiDAR point clouds.

## 1 Introduction

Forest inventory is a systematic process of collecting information essential for forest management, ecological monitoring, and sustainable resource planning [[1], [2], [3]]. Individual tree segmentation is a fundamental component of this process, as it enables the extraction of tree-level attributes such as tree height, crown diameter, crown volume, and biomass, which are critical for forest structure analysis and carbon stock estimation [[4], [5], [6], [7]]. Airborne and terrestrial LiDAR technologies can capture detailed three-dimensional forest structure from the canopy to the ground [8,9], making individual tree segmentation feasible in complex forest environments. However, accurate delineation remains highly challenging in practice due to dense crown overlap, multilayer canopy structures, and occlusion.

Early studies on individual tree segmentation from LiDAR data typically relied on top-view representations. In these methods, 3D point clouds were projected onto 2D canopy height models (CHMs), where potential treetops were detected using local maxima detection, variable window filtering, or template matching [10,11]. These treetop candidates were then used as seeds for crown delineation methods such as region growing or marker-controlled watershed segmentation [12,13]. Although CHM-based methods are computationally efficient and suitable for large-scale forest inventories, projecting three-dimensional point clouds into rasterized canopy representations inevitably discards structural information, particularly in dense forests where adjacent crowns frequently overlap.

To better exploit full 3D structural information, subsequent studies moved toward direct point-cloud processing using top-down and bottom-up strategies. Top-down methods begin from the canopy and group points into individual trees using cues such as tree height, treetop location, or predicted instance centers [[14], [15], [16]]. Bottom-up methods first identify more stable features such as trunks or roots, and then aggregate canopy points through upward expansion. Bottom-up approaches are often implemented through distance-based clustering, graph-cut analysis, or topology-aware association [[17], [18], [19], [20], [21], [22], [23]]. Compared with CHM-based methods, these strategies better preserve 3D structural details. However, their effectiveness still often depends on heuristic design and careful parameter tuning under specific site conditions, which limits robust generalization across different forest types.

These limitations have motivated the development of deep learning methods for individual tree segmentation from forest point clouds. Early studies adapted general point-cloud networks such as PointNet and PointNet++ for semantic segmentation or tree-component classification in forest scenes, providing a general feature extraction framework for learning-based semantic and instance modeling in forest point clouds [24,25]. However, in most existing methods, semantic supervision remains either coarse or flat, rather than being organized to reflect the hierarchical structure of trees. As a result, the learned representation does not explicitly capture how whole-tree identity relates to internal components such as woody parts and foliage, which limits discrimination in vertically stratified canopies and among neighboring trees with similar overall geometry.

Building on these backbones, methods such as TreeLearn formulate individual tree segmentation as a bottom-up point-level instance grouping problem, learning point embeddings or offsets to group points belonging to the same tree instance [26]. These embedding-based frameworks improve instance discrimination under crown overlap and trunk occlusion, but their grouping quality still depends heavily on local feature separability and often degrades in structurally complex stands. ForAINet further introduces joint semantic and instance learning with multi-category semantic supervision (e.g., ground, stems, branches) [27]. Subsequent studies enhance robustness through cross-platform training and prompt-based segmentation mechanisms [28,29]. More recently, Transformer architectures have been introduced to jointly model semantic and instance segmentation with attention mechanisms and query-based mask prediction [30].

Despite these advances, three key challenges remain. First, *lack of hierarchical semantic supervision*. Existing methods usually treat semantic labels as flat or coarse cues, rather than organizing them into multi-granularity supervision aligned with forest structure. As a result, the model cannot explicitly learn the part-whole semantics linking a tree to its internal components, such as woody components and foliage, which weakens representation learning in multilayer canopies and among neighboring trees with similar global geometry. Second, *weak semantic-instance coupling*. Even when semantic prediction and instance partition are learned jointly, they are typically optimized with separate objectives and without explicit consistency constraints. This weak coupling allows semantic labels and instance boundaries to conflict, such that semantically coherent tree components may be split across different instances, while a single instance may contain implausible semantic compositions. Third, *forest-specific cross-block ambiguity*. Large-scale forest inference usually relies on overlapping cropping followed by prediction merging, but in dense stands, boundary-crossing partial crowns and laterally overlapping trees make this process particularly error-prone. Existing merging strategies mainly rely on score ordering or geometric overlap, without explicitly modeling cross-block spatial correspondence and instance-level semantic agreement, so boundary crowns are prone to repeated instantiation or inconsistent identities. These limitations not only restrict further gains in segmentation accuracy, but also reduce the reliability of downstream tree-level measurements such as tree height, crown diameter, and crown volume, which are central to forest inventory and carbon assessment [31].

To address these challenges, we propose ForestSeg3D, a semantically guided framework for individual tree segmentation from forest LiDAR point clouds. The central idea of ForestSeg3D is to improve tree delineation consistently across representation learning and cross-task learning, while supporting robust large-scene inference in structurally complex forest environments. At the representation level, hierarchical semantic supervision (HSS) reformulates forest semantics as a coupled coarse-to-fine learning problem with explicit cross-level consistency, enabling the network to capture part-whole tree structure more effectively. At the learning level, bidirectional cross-task distillation (BCTD) explicitly bridges semantic prediction and instance partition, so that semantic cues regularize instance formation while tree regions promote semantic coherence. To support scene-level prediction in large forest plots, we further incorporate semantic-aware region merging (SRM), an inference-time consolidation strategy that extends overlap-based merging with instance-level semantic agreement to alleviate cross-block ambiguity caused by overlapping crowns and boundary-crossing partial trees. Together, these designs improve not only segmentation quality, but also the reliability of downstream tree-level structural measurements for large-area forest inventory.

Our main contributions are summarized as follows:

• Rather than treating semantics as flat auxiliary labels, we introduce HSS for individual tree segmentation. HSS couples coarse Tree/Non-Tree separation with fine-grained Ground/Wood/Leaf prediction through an explicit cross-level consistency constraint, enabling more effective learning of part-whole tree structure in multilayer and cluttered canopies.
• We introduce BCTD to explicitly bridge semantic prediction and instance partition. Semantic foreground cues regularize instance formation, while instance regions enforce within-tree semantic coherence, reducing conflicts between semantic labels and instance boundaries and leading to more coherent tree delineation.
• To support large-scale forest inference, we incorporate SRM, an inference-time scene-level consolidation strategy that extends overlap-based merging with instance-level semantic agreement. SRM reduces duplicated or conflicting predictions caused by overlapping crowns and boundary-crossing trees, thereby improving scene-level consistency for large-area forest inventory.

## 2 Materials and methods

### 2.1 Study data

We evaluated the proposed method on two publicly available forest point cloud datasets, FOR-instanceV2 [30] and ForestSemantic [32]. These datasets differ in forest conditions, acquisition platforms, and annotation characteristics across diverse geographic regions, providing a representative evaluation setting for forest inventory and structural assessment applications.

#### 2.1.1 FOR-instanceV2 dataset

FOR-instanceV2 is an extended large-scale benchmark dataset for forest point cloud segmentation. It extends the original FOR-instance dataset [33] by incorporating additional challenging datasets collected from multiple geographic regions, including Europe, Oceania, and Asia. The dataset covers a wide range of forest types, including boreal, temperate, and tropical forests, with diverse tree species and structural characteristics. The original FOR-instance benchmark comprises five UAV laser scanning collections from different countries and provides manually annotated instance- and semantic-level labels for individual trees and major forest components. The dataset integrates point clouds acquired using multiple sensing platforms, including unmanned aerial laser scanning (ULS), mobile laser scanning (MLS), and terrestrial laser scanning (TLS), leading to significant variability in point density, acquisition geometry, and viewing perspectives. Compared with the original dataset, FOR-instanceV2 increases the total number of annotated individual trees approximately eightfold through the inclusion of multiple newly collected and previously unavailable forest datasets [30]. It provides both instance-level labels and semantic labels for ground, wood, and leaf. Owing to its large scale and high diversity, FOR-instanceV2 is used as the main dataset for segmentation evaluation. Structural parameters are computed from point clouds using the same algorithm for predicted instances and manually annotated ground-truth (GT) point-cloud instances. Parameters from GT point-cloud instances are used as reference values for comparison. This design is adopted because structural attributes are not publicly available in the dataset.

#### 2.1.2 ForestSemantic dataset

ForestSemantic is a high-resolution forest point cloud dataset acquired using TLS. It contains six boreal forest plots located in Evo, Finland, representing mixed stands of Scots pine, Norway spruce, and birch. The plots are categorized into easy, medium, and difficult levels according to stand density. According to the dataset description, the mean stem density increases from approximately 750 stems ha^−1^ in easy plots to over 2000 stems ha^−1^ in difficult plots. In this study, we use the three publicly available plots, namely Plot 1, Plot 3, and Plot 5, which represent the easy, medium, and difficult categories, respectively. ForestSemantic provides both instance-level and semantic annotations for detailed forest scene analysis. Compared with FOR-instanceV2, it provides denser TLS point clouds and more controlled structural variation, and is therefore used as a supplementary dataset to evaluate the robustness of segmentation performance under different forest conditions.

### 2.2 Data preprocessing

To handle large-scale forest scenes while preserving geometric details, we adopt a two-stage preprocessing strategy consisting of scene-level redundancy reduction and crop-level adaptive sampling.

*Scene-level preprocessing*. At the scene level, raw point clouds are first downsampled using a voxel size of 0.1 m to remove redundant points and improve storage and loading efficiency [26]. This step is applied once offline before training and inference. Coordinates are then normalized by subtracting the per-scene horizontal mean, computed as the arithmetic mean of the x- and y-coordinates of all points in the scene, while the vertical coordinate is normalized by subtracting the minimum z-coordinate, thereby centering each scene in the horizontal plane and aligning it to zero elevation.

*Crop generation*. During training, we employ adaptive cylindrical cropping to construct input samples. Cylindrical regions are sampled with a radius in the range of [6.0 m, 12.0 m], and for each sampled center the radius is iteratively adjusted using binary search for up to 5 iterations to make the pre-voxelization crop size close to 80, 000 points. This point budget serves as an upper limit before online voxelization to balance spatial coverage and GPU memory, rather than the exact number of points fed into the network. The lower bound of 6.0 m helps ensure that each crop contains a sufficient number of tree instances, while the upper bound of 12.0 m limits memory consumption and remains consistent in scale with prior forest segmentation frameworks [30]. Crop centers are randomly sampled from the scene. GT instances intersecting a crop are retained without additional filtering for boundary truncation, allowing the model to learn under partially observed tree conditions that are also encountered during sliding-window inference. Data augmentation includes random rotation around the vertical axis within [−*π*, *π*], random scaling in [0.8, 1.2], and random translation with standard deviation 0.1 m.

*Point sampling and voxelization*. After cropping, an online voxel-based sampling step with a voxel grid of 0.08 m is applied to regularize the crop-level point distribution and reduce local density variation. Random jitter is further applied to the voxel origin as data augmentation, which improves sampling diversity before sparse quantization. The sampled points are then quantized into sparse tensors with a voxel resolution of 0.12 m for the backbone, where the 0.08 m grid controls crop-level sampling density and the 0.12 m grid defines the sparse-convolution input resolution. Superpoints are generated by graph-based geometric over-segmentation [34] and serve as the basic processing units for feature aggregation and query decoding.

### 2.3 Methodology

#### 2.3.1 Overall architecture

**Fig. 1:**
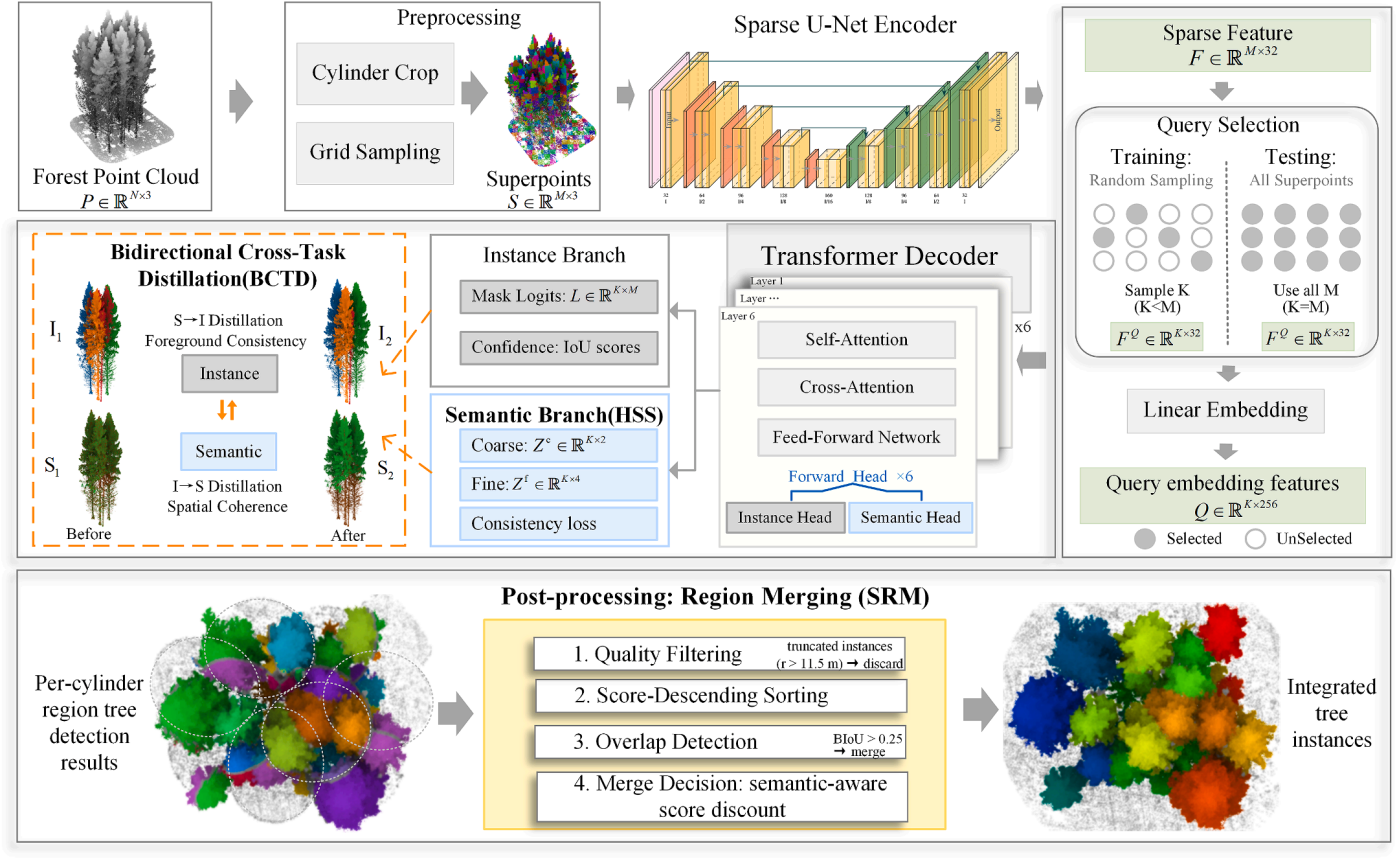
Overall architecture of ForestSeg3D. The framework consists of two core learning modules, HSS and BCTD, together with an inference-time semantic-aware region merging (SRM) strategy. Forest point clouds are partitioned into superpoints and encoded by a sparse U-Net backbone. Query embeddings are refined by a Transformer decoder to predict instance masks and hierarchical semantic labels. HSS provides coarse-to-fine semantic supervision with cross-level consistency, while BCTD explicitly couples semantic and instance learning through foreground consistency and spatial coherence. During inference, SRM consolidates crop-level predictions into scene-level tree instances by combining overlap-based association with instance-level semantic agreement.

As illustrated in Fig. 1, ForestSeg3D adopts a query-based encoder–decoder architecture for individual tree segmentation from forest point clouds. Given an input point cloud $P\in R^{N\times 3}$, cylindrical cropping and voxel/grid sampling are first applied to regularize the input points, after which graph-based over-segmentation is used to generate superpoints as the basic processing units for subsequent sparse convolution and query-based decoding. A sparse U-Net encoder extracts multi-scale geometric features from the voxelized point cloud, which are then aggregated into superpoint-level representations $F\in R^{M\times 32}$, where *M* is the number of superpoints.

Based on the encoded superpoint features, a set of query embeddings $Q\in R^{K\times 256}$ is constructed and fed into a 6-layer Transformer decoder. Through repeated self-attention and cross-attention, the decoder progressively refines the query features and predicts query-wise instance masks and semantic labels. The instance branch outputs mask logits $L\in R^{K\times M}$ together with confidence scores for individual tree segmentation, while the semantic branch produces hierarchical semantic predictions, including a coarse Tree/Non-Tree partition and a fine-grained semantic component classification over Ground, Wood, and Leaf.

On top of this encoder–decoder framework, ForestSeg3D incorporates two learning modules and one inference-time consolidation strategy. First, HSS imposes coarse-to-fine semantic constraints on the shared query features, encouraging them to encode both global scene structure and fine-grained tree-component cues. Second, BCTD explicitly couples the semantic and instance branches by enforcing foreground consistency for predicted instance masks and spatial coherence for semantic predictions within tree regions. During inference, large forest scenes are processed using overlapping cylindrical regions for memory efficiency, and SRM consolidates per-region predictions through quality filtering, overlap-aware association, and semantic-aware overlap adjustment to reduce duplicated or conflicting tree instances across region boundaries.

#### 2.3.2 Hierarchical semantic supervision

Forest point clouds exhibit an inherent semantic hierarchy. At a coarse level, Tree and Non-Tree regions, corresponding to Wood/Leaf and Ground in our annotation space, are relatively easy to separate because they differ substantially in elevation distribution and structural organization. At a finer level, however, distinguishing Wood from Leaf is more challenging due to their close spatial adjacency and frequent overlap within the same crown. Although instance supervision alone can guide individual tree segmentation, it does not explicitly encourage the shared query features to capture such multi-level semantic structure. In particular, the coarse Tree/Non-Tree separation provides a global structural prior that suppresses ground interference near stem bases, while the fine Wood/Leaf distinction provides component-level cues for preserving crown and stem structure. Without such explicit coupling, instance supervision alone cannot reliably determine whether an ambiguous mask boundary corresponds to a true tree edge, a ground–vegetation transition, or a contact region between neighboring crowns.

To address this issue, we introduce HSS, which decomposes semantic learning into a coarse Tree/Non-Tree separation task and a fine-grained semantic component classification task targeting Ground, Wood, and Leaf, as illustrated by the semantic branch in Fig. 1. In our formulation, the fine branch predicts a complete three-class semantic partition over Ground, Wood, and Leaf. The coarse branch, in contrast, operates on binary labels derived from the fine-grained GT annotations, where Ground is mapped to Non-Tree and Wood and Leaf are mapped to Tree. Therefore, the two branches are related through a hierarchical consistency constraint rather than a strict tree-structured label decomposition.

Specifically, two MLP heads are attached to the shared query features of *K* queries. The coarse head predicts logits $Z^{c}\in R^{K\times 2}$ for Tree/Non-Tree separation, and the fine head predicts logits $Z^{f}\in R^{K\times \left(C+1\right)}$ for semantic component classification, where *C* = 3 denotes Ground, Wood, and Leaf. The extra channel in **Z**^*f*^ is an auxiliary background class used to absorb unmatched or ambiguous queries during training.

The coarse and fine semantic probabilities are defined as

(1) $$P^{c}=softmax\left(Z^{c}\right)\in R^{K\times 2},$$

(2) $$P^{f,train}=softmax\left(Z^{f}\right)\in R^{K\times \left(C+1\right)}.$$

During inference, the auxiliary background channel is excluded:

(3) $$P^{f}=softmax\left(Z_{:,\;:C}^{f}\right)\in R^{K\times C}.$$

We use focal loss for both the coarse and fine semantic branches to mitigate class imbalance, with class-frequency-based weighting for the fine-grained semantic categories.

To couple the two semantic levels, we further impose a hierarchical consistency constraint:

(4) $$L_{\text{cons}}=\frac{1}{K}\overset{K}{\underset{k=1}{\sum }}{\left(P_{k,\;Tree}^{c}-P_{k,\;Wood}^{f,train}-P_{k,\;Leaf}^{f,train}\right)}^{2},$$

where $P_{k,\;Tree}^{c}$ denotes the Tree probability from the coarse branch. This formulation is adopted because the coarse Tree category corresponds to the union of Wood and Leaf in the fine-grained label space, whereas Ground is mapped to the complementary class. Accordingly, this term encourages the fine predictions to remain compatible with the coarse Tree/Non-Tree partition and reduces contradictory supervision between the two semantic heads.

The overall HSS objective is defined as

(5) $$L_{\text{HSS}}=\lambda_{1}\left(e\right)L_{\text{coarse}}+\lambda_{2}\left(e\right)L_{\text{fine}}+\alpha_{3}L_{\text{cons}},$$

where *λ*_1_(*e*) and *λ*_2_(*e*) follow a fixed progressive schedule over training epochs, assigning stronger emphasis to the coarse branch at early stages and gradually increasing the contribution of the fine branch as the shared representation becomes more discriminative. The base weights and schedule details are provided in the Supplementary Materials. The hierarchical consistency weight is fixed to *α*_3_ = 0.5, which is justified by the sensitivity analysis reported in Supplementary Fig. S2.

#### 2.3.3 Bidirectional cross-task distillation

While HSS provides structured semantic guidance, the instance and semantic branches are still optimized through separate objectives. In cluttered forest scenes where crowns intertwine, instance masks may extend into semantically implausible regions such as ground, while semantic predictions may exhibit unnecessary local fluctuation within the same tree region. To explicitly couple the two tasks, we introduce BCTD, as shown in Fig. 1. The semantic branch constrains the spatial extent of instance masks, and instance regions in turn regularize semantic coherence.

To bridge query-level and superpoint-level representations, each superpoint is assigned to the query with the highest mask response:

(6) $$k^{*}\left(s\right)=\arg\max_{k}\sigma \left(l_{ks}\right),$$

where *l*_*ks*_ is the mask logit of query *k* at superpoint *s*, and *σ*(⋅) is the sigmoid function. The corresponding superpoint-level fine semantic probability is then defined as

(7) $${\hat{P}}_{s}^{f}\left(c\right)=P_{k^{*}\left(s\right),\;c}^{f}.$$

*Semantic-to-Instance: Foreground consistency loss*. For each predicted instance mask $l_{k}\in R^{M}$, we define the semantic foreground probability at superpoint *s* as

(8) $$p_{s}^{\text{fg}}={\hat{P}}_{s}^{f}\left(Wood\right)+{\hat{P}}_{s}^{f}\left(Leaf\right).$$

The foreground consistency loss is formulated as

(9) $$L_{S\to I}=\frac{1}{K}\overset{K}{\underset{k=1}{\sum }}{\left(1-\frac{\sum_{s=1}^{M}\sigma \left(l_{ks}\right)\;p_{s}^{\text{fg}}}{\sum_{s=1}^{M}\sigma \left(l_{ks}\right)+\epsilon }\right)}^{2},$$

where *ϵ* is a small constant for numerical stability. This term anchors instance extent to semantically plausible tree foreground and suppresses mask leakage into ground regions.

Instance-to-Semantic: Spatial coherence loss. Using GT instance masks as spatial supports, we minimize semantic variance among high-confidence superpoints within each tree region. Let $S_{k}=\left\{s|l_{k}^{\text{gt}}\left(s\right)=1,\;Conf\left({\hat{P}}_{s}^{f}\right)>\tau \right\}$ denote the set of high-confidence superpoints in the *k*-th GT instance, where $Conf\left({\hat{P}}_{s}^{f}\right)=\max_{c}{\hat{P}}_{s}^{f}\left(c\right)$ and *τ* = 0.6. The mean semantic distribution within this set is

(10) $${\bar{P}}_{k}^{f}=\frac{1}{\left|S_{k}\right|}\underset{s\in S_{k}}{\sum }{\hat{P}}_{s}^{f},$$

and the spatial coherence loss is defined as

(11) $$L_{I\to S}=\frac{1}{K^{\text{gt}}}\overset{K^{\text{gt}}}{\underset{k=1}{\sum }}\sqrt{\frac{1}{\left|S_{k}\right|}\underset{s\in S_{k}}{\sum }\|{\hat{P}}_{s}^{f}-{\bar{P}}_{k}^{f}{\|}^{2}+\epsilon }.$$

This term enforces within-instance semantic coherence and discourages fragmented semantic predictions that would otherwise destabilize instance boundary localization. Instances with empty $S_{k}$ are excluded from the computation.

The complete bidirectional distillation loss is

(12) $$L_{\text{distill}}=\lambda_{S\to I}^{\text{eff}}L_{S\to I}+\lambda_{I\to S}^{\text{eff}}L_{I\to S},$$

where $\lambda_{S\to I}^{\text{eff}}=1.2$ and $\lambda_{I\to S}^{\text{eff}}=0.675$ denote the final effective weights of the two distillation directions. These values correspond to the combined scaling used in implementation, with stronger emphasis on semantic-to-instance regularization.

By explicitly coupling semantic plausibility and instance coherence, BCTD encourages the instance branch to avoid semantically implausible regions while promoting smoother semantic organization within each tree, reducing cross-instance confusion in crowded forest scenes.

### 2.4 Training objectives and large-scale inference

*Instance segmentation loss*. Following standard practice in unified segmentation [35], the instance loss combines classification, binary mask, dice, and IoU components:

(13) $$L_{\text{inst}}=\beta_{1}L_{\text{cls}}+\beta_{2}L_{\text{mask}}+\beta_{3}L_{\text{dice}}+\beta_{4}L_{\text{IoU}},$$

where *β*_1_ = 0.5, *β*_2_ = 3.0, *β*_3_ = 3.0, and *β*_4_ = 0.7 follow standard settings in prior unified segmentation frameworks. Instance-to-GT matching uses the Hungarian algorithm with classification, mask BCE, and dice loss.

*Overall training objective*. Our complete training objective integrates instance segmentation loss, hierarchical semantic supervision, and bidirectional distillation:

(14) $$L_{\text{total}}=\lambda_{\text{inst}}L_{\text{inst}}+\lambda_{\text{sem}}L_{\text{HSS}}+L_{\text{distill}},$$

where *λ*_inst_ = 4.0 and *λ*_sem_ = 1.0 prioritize the instance objective while incorporating semantic and distillation guidance.

*Semantic-aware region merging (SRM)*. For memory-efficient inference on large forest scenes, each scene is partitioned into overlapping cylindrical regions and processed independently. Inference crops use a fixed cylindrical radius of 12.0 m, with crop centers placed on a regular grid at a step size of 3.0 m, yielding substantial overlap between neighboring regions. This high-overlap design ensures robust coverage of boundary areas, where trees frequently span multiple crops in dense forest scenes. However, it also introduces a forest-specific consolidation challenge: boundary-crossing crowns and laterally overlapping trees may produce partial, duplicated, or conflicting predictions across neighboring crops. Therefore, scene-level consolidation must not only suppress redundant masks, but also determine whether spatially overlapping predictions are semantically consistent instance hypotheses.

Accordingly, we adopt a semantic-aware scene-level consolidation strategy that jointly leverages spatial correspondence and instance-level semantic agreement, rather than relying only on score ordering and geometric overlap. Semantic predictions from overlapping crops are aggregated by per-point majority voting across all crops. For instance predictions, candidate masks with a confidence score below 0.018 or fewer than 15 points are first discarded; an additional edge-proximity filter then removes instances extending beyond 11.5 m from the crop center, corresponding to a 0.5 m margin inside the cylindrical boundary, which suppresses severely truncated predictions. During merging, the remaining candidates are processed in descending score order, and their overlap with existing scene-level instances is evaluated using the bidirectional IoU (BIoU):

(15) $$BIoU\left(A,B\right)=\max\;\left(\frac{\left|A\cap B\right|}{\left|A\right|},\frac{\left|A\cap B\right|}{\left|B\right|}\right),$$

where *A* and *B* denote the point sets of two candidate instance masks. Compared with standard IoU, this formulation is better suited to forest scenes containing trees of highly variable sizes, because it is more tolerant of partial overlap between instances with different spatial extents. To reduce incorrect merging between spatially overlapping but semantically inconsistent regions, the computed BIoU is discounted by a fixed factor of 0.5 when the predicted instance-level semantic labels of two candidate instances disagree, where each instance label is taken as the argmax of the corresponding query classification head output. In this way, the overlap-based association is further conditioned on semantic agreement between candidate instances. Candidate instances are merged using an overlap threshold *τ*_merge_ = 0.25, and its sensitivity is further analyzed in Supplementary Fig. S3.

## 3 Experiments

### 3.1 Experimental setup

#### 3.1.1 Experimental setting

We implement ForestSeg3D based on UniSeg3D [35] and compare it with traditional and graph-based methods, including OSC [23] and GOT [22]; forest-specific methods, including ForAINet [27], 3DPSNet [29], TreeLearn [26], SegmentAnyTree [28], and ForestFormer3D [30]; and the generic framework OneFormer3D [36]. Experiments are conducted on FOR-instanceV2 using the official train/validation/test split and on ForestSemantic using 3-fold leave-one-plot-out cross-validation over the three publicly available plots, namely Plot 1, Plot 3, and Plot 5. In each fold of ForestSemantic, two plots are used for training and the held-out plot is used for evaluation. The overall ForestSemantic results are computed by globally aggregating TP, FP, FN, and GT over the three held-out folds.

All learning-based baselines are re-trained and adapted to FOR-instanceV2. Method-specific hyperparameters, such as crop size, training epochs, and learning rate, are adjusted following their original design principles, while keeping the core architectures and training pipelines consistent. Unless otherwise specified, all methods share the same data preprocessing and evaluation pipeline. Detailed baseline configurations, ForestSeg3D hyperparameter settings, and ForestSemantic cross-validation splits are provided in Supplementary Sections S3–S5 and Supplementary Table S1–S2. ForestSeg3D is trained for 250 epochs using AdamW with an initial learning rate of 10^−4^ and a cosine annealing schedule. All experiments are implemented in Python 3.10 with PyTorch 1.13.0 and CUDA 11.7, and conducted on an NVIDIA L40S GPU.

The BlueCat region in FOR-instanceV2 is excluded from both quantitative and qualitative evaluations because it predominantly contains shrubs and juvenile trees rather than mature trees, which are the primary focus of this study. Specifically, although the region includes 537 annotated instances, 76.5% of them are lower than 5 m, with a median height of only 2.2 m. This distribution differs substantially from the other regions in FOR-instanceV2, which are dominated by mature trees with well-defined crowns. Including BlueCat would therefore introduce a mismatch in object definition and bias the comparison across methods.

### 3.2 Evaluation metrics

Following prior studies [28,30], predicted tree instances were matched to GT instances using IoU-based instance matching. At an IoU threshold of 0.5, a prediction was counted as a true positive (TP) if it matched a GT instance with IoU ≥0.5; otherwise, unmatched predictions and GT instances were counted as false positives (FP) and false negatives (FN), respectively. Based on these matches, we computed Detection, Omission, and Commission as:

(16) $$\begin{array}{l} \text{Detection}=\frac{TP}{GT},\;\text{Omission}=\frac{FN}{GT},\\ \text{Commission}=\frac{FP}{TP+FP} \end{array}$$

All segmentation metrics are computed globally by aggregating TP, FP, FN, and GT over the corresponding evaluation set before calculating the final scores. We further report the global F1-score to summarize the overall instance segmentation quality:

(17) $$\text{F}1=\frac{2\times \text{Precision}\times \text{Recall}}{\text{Precision}+\text{Recall}},$$

where

(18) $$\text{Precision}=\frac{TP}{TP+FP},\;\text{Recall}=\frac{TP}{TP+FN}.$$

To evaluate crown delineation under different localization strictness levels, we compute Average Precision (AP) at IoU thresholds of 0.25, 0.50, and 0.75. The mean Average Precision (mAP) is defined as:

(19) $$\text{mAP}=\frac{\text{AP@}0.25+\text{AP@}0.50+\text{AP@}0.75}{3}.$$

In addition to segmentation accuracy, we further evaluate individual-tree structural traits using correctly matched tree pairs (i.e., TPs at IoU = 0.5). The evaluated traits include tree height (*H*), crown diameter (*CD*), and crown volume (*CV*). Tree height is defined as the vertical distance between the tree top and the local ground reference. Crown diameter is defined as the maximum horizontal span of the crown in the *XY* plane. Crown volume is estimated by voxelizing the crown point cloud at a resolution of *r* = 0.2 m, where crown points are defined as leaf points together with the upper 50% of wood points, after removing isolated outliers using HDBSCAN [37]. The volume is then computed as *CV* = *N*_*v*_ ⋅ *r*^3^, where *N*_*v*_ denotes the number of occupied voxels.

For structural parameter estimation, let $y_{i}^{\mathrm{ref}}$ and $y_{i}^{\mathrm{pred}}$ denote the reference and predicted values of a given trait for the *i*-th matched tree pair. For tree height and crown diameter, the estimation error is quantified using the root mean square error (RMSE):

(20) $$\text{RMSE}\left(y\right)=\sqrt{\frac{1}{N}\overset{N}{\underset{i=1}{\sum }}{\left(y_{i}^{\mathrm{ref}}-y_{i}^{\mathrm{pred}}\right)}^{2}},$$

where *N* is the number of matched trees and *y* ∈ {*H*, *CD*}.

To further evaluate the agreement between predicted and reference structural traits, we calculate the coefficient of determination (*R*^2^):

(21) $$R^{2}=1-\frac{\overset{N}{\underset{i=1}{\sum }}{\left(y_{i}^{\mathrm{ref}}-y_{i}^{\mathrm{pred}}\right)}^{2}}{\overset{N}{\underset{i=1}{\sum }}{\left(y_{i}^{\mathrm{ref}}-{\bar{y}}^{\mathrm{ref}}\right)}^{2}},$$

where ${\bar{y}}^{\mathrm{ref}}$ denotes the mean of the reference values.

### 3.3 Comparison with state-of-the-art methods

#### 3.3.1 Results on FOR-instanceV2

Table 1 reports the quantitative comparison on the FOR-instanceV2 test set. ForestSeg3D achieves the best overall trade-off on FOR-instanceV2, with the highest Detection rate of 80.98%, the lowest Omission of 19.02%, the lowest Commission of 7.50%, the highest F1-score of 86.36%, and a competitive second-best mAP of 73.25%. Compared with the baseline UniSeg3D, ForestSeg3D improves Detection by 9.52 percentage points (pp), reduces Omission by 9.52 pp, reduces Commission by 5.06 pp, improves F1-score by 7.71 pp, and improves mAP by 10.94 pp. Compared with ForestFormer3D, our method further improves Detection by 1.76 pp, reduces Omission by 1.76 pp, reduces Commission by 0.42 pp, and improves F1-score by 1.19 pp, while maintaining a comparable but slightly lower mAP (73.25% vs. 73.63%).

**Table 1:** Performance comparison on FOR-instanceV2 test set (%). Best results in **bold**, second best underlined. OSC: Oriented Search and Clustering, GOT: Graph Optimization, OF3D: OneFormer3D, SAT: SegmentAnyTree, FF3D: ForestFormer3D. †: GT-guided prompt mode.

Region	Metric(%)	OSC [23]	GOT [22]	ForAINet [27]	3DPSNet^†^ [29]	TreeLearn [26]	OF3D [36]	SAT [28]	UniSeg3D [35]	FF3D [30]	**Ours**
CULS	Det *↑*	90.00	**100.00**	95.24	**100.00**	**100.00**	90.48	95.24	**100.00**	95.24	**100.00**
	Omi *↓*	10.00	**0.00**	4.76	**0.00**	**0.00**	9.52	4.76	**0.00**	4.76	**0.00**
	Com *↓*	18.18	4.76	**0.00**	**0.00**	44.44	5.00	28.57	**0.00**	**0.00**	**0.00**
	F1 *↑*	85.71	97.56	97.56	**100.00**	71.43	92.68	81.63	**100.00**	97.56	**100.00**
	mAP *↑*	86.94	86.90	92.06	93.33	71.40	76.48	77.30	**100.00**	90.91	**100.00**
NIBIO	Det *↑*	46.58	32.30	67.66	81.37	**95.03**	56.29	85.03	76.92	87.43	89.54
	Omi *↓*	53.42	67.70	32.34	18.63	**4.97**	43.71	14.97	23.08	12.57	10.46
	Com *↓*	21.05	66.67	14.39	12.67	80.78	24.80	14.97	10.91	**4.58**	5.08
	F1 *↑*	58.59	32.81	75.59	84.24	31.97	64.38	85.03	82.36	91.25	**91.97**
	mAP *↑*	38.45	25.38	65.33	77.56	32.30	52.92	72.09	68.03	81.85	**84.12**
RMIT	Det *↑*	38.33	33.33	58.46	35.94	53.12	55.38	67.69	56.67	**78.46**	73.33
	Omi *↓*	61.67	66.67	41.54	64.06	46.88	44.62	32.31	43.33	**21.54**	26.67
	Com *↓*	36.11	56.52	22.45	34.29	52.78	33.33	21.43	20.93	10.53	**6.38**
	F1 *↑*	47.92	37.74	66.67	46.46	50.00	60.50	72.73	66.02	**83.61**	82.24
	mAP *↑*	26.81	22.12	54.36	34.89	49.00	45.80	49.18	48.06	**69.41**	63.88
SCION	Det *↑*	72.09	51.16	75.56	81.40	**95.35**	46.67	86.67	78.89	91.11	91.22
	Omi *↓*	27.91	48.84	24.44	18.60	**4.65**	53.33	13.33	21.11	8.89	8.78
	Com *↓*	47.46	48.84	**0.00**	18.60	87.87	12.50	27.78	13.37	**0.00**	11.26
	F1 *↑*	60.78	51.16	86.08	81.40	21.52	60.87	78.79	82.56	**95.35**	89.95
	mAP *↑*	62.51	44.12	71.63	73.19	21.40	40.92	70.23	73.66	82.39	**88.21**
TUWIEN	Det *↑*	17.14	31.43	41.67	45.71	**60.00**	27.78	58.33	42.86	58.33	**60.00**
	Omi *↓*	82.86	68.57	58.33	54.29	**40.00**	72.22	41.67	57.14	41.67	**40.00**
	Com *↓*	76.92	64.52	48.28	51.52	88.27	79.59	43.24	62.50	**27.59**	44.74
	F1 *↑*	19.67	33.33	46.15	47.06	19.63	23.53	57.53	40.00	**64.62**	57.53
	mAP *↑*	14.80	26.66	38.89	40.95	19.90	18.98	38.17	26.47	**46.38**	37.69
NIBIO2	Det *↑*	36.62	36.98	60.80	49.34	63.08	61.41	73.71	74.12	77.62	**82.03**
	Omi *↓*	63.38	63.02	39.20	50.66	36.92	38.59	26.29	25.88	22.38	**17.97**
	Com *↓*	24.75	55.03	16.99	15.20	62.50	22.55	17.15	8.86	8.47	**5.31**
	F1 *↑*	49.26	40.59	70.19	62.39	47.04	68.50	78.01	81.18	84.00	**87.48**
	mAP *↑*	27.00	29.63	60.20	47.13	45.29	52.79	62.21	66.16	70.43	**74.28**
NIBIO_MLS	Det *↑*	56.52	86.96	75.00	34.78	**95.65**	62.50	91.67	78.26	91.67	91.30
	Omi *↓*	43.48	13.04	25.00	65.22	**4.35**	37.50	8.33	21.74	8.33	8.70
	Com *↓*	27.78	13.04	21.74	20.00	78.85	16.67	21.43	5.26	**0.00**	4.55
	F1 *↑*	63.41	86.96	76.60	48.48	34.65	71.43	84.62	85.71	**95.65**	93.33
	mAP *↑*	48.22	73.92	68.06	28.99	33.60	54.55	70.68	70.49	**87.60**	83.55
Yuchen	Det *↑*	50.00	20.83	64.00	-	-	56.00	**76.00**	54.17	64.00	70.83
	Omi *↓*	50.00	79.17	36.00	-	-	44.00	**24.00**	45.83	36.00	29.17
	Com *↓*	55.56	28.57	33.33	-	-	22.22	36.67	43.48	**11.11**	19.05
	F1 *↑*	47.06	32.26	65.31	-	-	65.12	69.09	55.32	74.42	**75.56**
	mAP *↑*	32.15	16.90	**69.33**	-	-	39.96	49.42	49.43	55.09	61.30
**Overall**	Det *↑*	40.32	38.22	62.51	54.61	69.23	59.29	75.71	71.46	79.22	**80.98**
	Omi *↓*	59.68	61.78	37.49	45.39	30.77	40.71	24.29	28.54	20.78	**19.02**
	Com *↓*	29.87	54.74	17.43	16.97	72.08	25.56	19.26	12.56	7.92	**7.50**
	F1 *↑*	51.20	41.44	71.15	65.88	39.80	66.00	78.14	78.65	85.17	**86.36**
	mAP *↑*	28.28	32.55	62.89	56.18	40.37	50.96	63.96	62.31	**73.63**	73.25

The per-region results further show that the proposed method performs consistently well across regions with different forest structures. In NIBIO2, ForestSeg3D achieves the best performance across all five metrics, reaching 82.03% Detection, 17.97% Omission, 5.31% Commission, 87.48% F1-score, and 74.28% mAP. In NIBIO, our method obtains the best F1-score and mAP, reaching 91.97% and 84.12%, while remaining highly competitive in Detection and Commission. In CULS, our method reaches perfect scores on Detection, Omission, Commission, F1-score, and mAP, tied with the best-performing methods. In Yuchen, ForestSeg3D achieves the best F1-score at 75.56%. TreeLearn and 3DPSNet do not produce valid predictions in this region under the current setting, which is likely related to their reliance on stem-aware or prompt-based instance initialization that becomes unstable under extremely sparse stem conditions.

In challenging regions including TUWIEN, SCION, and NIBIO_MLS, our method remains consistently among the top-performing approaches. In RMIT, however, ForestFormer3D achieves notably higher Detection (78.46% vs. 73.33%), suggesting that its design may be more favorable in relatively sparse yet structurally irregular plots. Overall, these results indicate that ForestSeg3D provides a well-balanced trade-off across diverse forest conditions, improving tree detection completeness while maintaining effective false-positive suppression, particularly in regions with stronger canopy interaction and more complex vertical structure.

#### 3.3.2 Results on ForestSemantic

Table 2 presents the comparison on the ForestSemantic dataset. ForestSeg3D achieves the best overall performance in Detection, Omission, F1-score, and mAP, with values of 80.79%, 19.21%, 80.40%, and 67.00%, respectively, while ranking second in Commission (20.00%), slightly behind SegmentAnyTree (15.04%). Compared with UniSeg3D, our method improves Detection by 12.91 pp, F1-score by 12.07 pp, and mAP by 14.00 pp. It also outperforms SegmentAnyTree in Detection, F1-score, and mAP, indicating a better balance between detection completeness and segmentation quality. These improvements indicate that the proposed semantic guidance and semantic-instance coupling remain effective when transferred to TLS forest plots with different stand densities.

**Table 2:** Performance comparison on ForestSemantic dataset. All metrics in %. Best results in **bold**, second best underlined. OSC: Oriented Search and Clustering, GOT: Graph Optimization, SAT: SegmentAnyTree, FF3D: ForestFormer3D. †: GT-guided prompt mode.

Method	Plot 1					Plot 3					Plot 5					Overall				
	Det *↑*	Omi *↓*	Com *↓*	F1 *↑*	mAP *↑*	Det *↑*	Omi *↓*	Com *↓*	F1 *↑*	mAP *↑*	Det *↑*	Omi *↓*	Com *↓*	F1 *↑*	mAP *↑*	Det *↑*	Omi *↓*	Com *↓*	F1 *↑*	mAP *↑*
OSC [23]	66.67	33.33	20.93	72.34	50.68	20.44	79.56	68.54	24.77	15.71	9.38	90.62	92.04	8.62	9.75	25.00	75.00	71.02	26.84	15.17
GOT [22]	47.06	52.94	52.94	47.06	39.99	29.93	70.07	70.29	29.82	25.33	20.83	79.17	81.98	19.32	17.48	29.93	70.07	71.67	29.11	27.60
3DPSNet^†^ [29]	84.62	15.38	4.35	89.80	68.59	61.43	38.57	**13.13**	71.97	53.81	33.90	66.10	52.38	39.60	31.92	59.98	40.02	23.29	67.12	51.44
TreeLearn [26]	70.60	29.40	26.50	72.00	74.00	52.90	47.10	19.60	63.80	59.20	44.40	55.60	33.30	53.30	48.90	52.60	47.40	26.00	61.50	60.70
SAT [28]	**98.04**	**1.96**	13.79	91.74	90.91	66.19	33.81	16.36	73.90	60.61	**71.79**	**28.21**	**14.29**	**78.14**	**60.61**	73.62	26.38	**15.04**	78.88	66.04
UniSeg3D [35]	**98.04**	**1.96**	3.85	97.09	95.79	68.61	31.39	14.55	76.11	72.87	53.51	46.49	55.15	48.80	44.00	67.88	32.12	31.21	68.33	53.00
FF3D [30]	88.24	11.76	6.25	93.75	81.82	44.60	55.40	29.55	70.47	45.45	19.66	80.34	57.41	33.33	18.18	43.00	57.00	27.87	53.88	40.52

**Ours**	**98.04**	**1.96**	**0.00**	**99.01**	**96.37**	**89.05**	**10.95**	13.48	**87.77**	**81.06**	63.16	36.84	36.84	63.16	55.85	**80.79**	**19.21**	20.00	**80.40**	**67.00**

The plot-wise results show that ForestSeg3D generalizes well across different stand conditions. On Plot 3, which represents a medium-density plot with more complex structures than Plot 1, it achieves the best Detection, Omission, F1-score, and mAP, with values of 89.05%, 10.95%, 87.77%, and 81.06%, respectively, while obtaining the second-best Commission. On Plot 1, it obtains the best Commission, F1-score, and mAP while tying for the best Detection. On Plot 5, ForestSeg3D remains competitive, achieving the second-best Detection, Omission, F1-score, and mAP, although its higher Commission indicates that false-positive control remains challenging in more complex scenes. This degradation is consistent with the difficult nature of Plot 5, where higher stand density and stronger vertical overlap can increase ambiguity between adjacent tree instances.

#### 3.3.3 Qualitative analysis

**Fig. 2:**
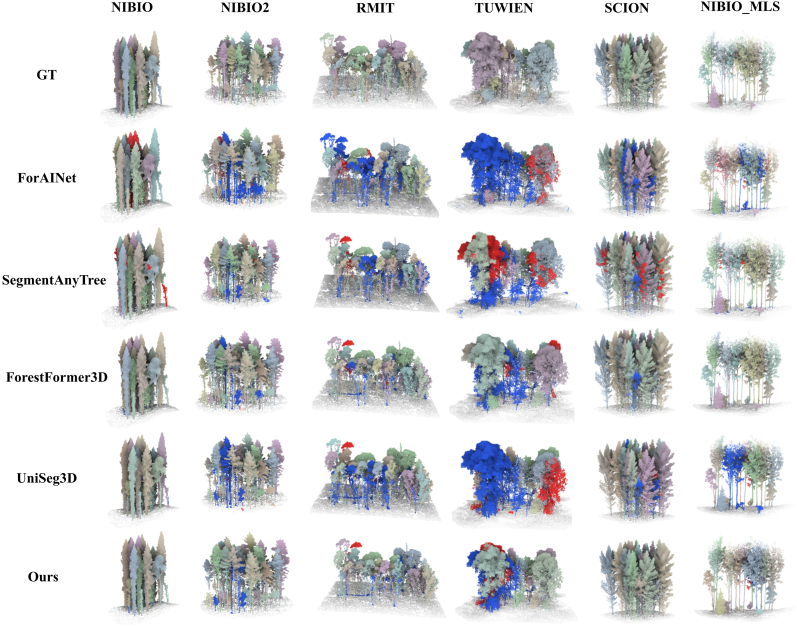
Qualitative comparison of different methods across six representative forest regions. Rows show the GT and predictions from different methods, while columns correspond to different forest regions. Red denotes false positives, and blue denotes false negatives with respect to the GT.

**Fig. 3:**
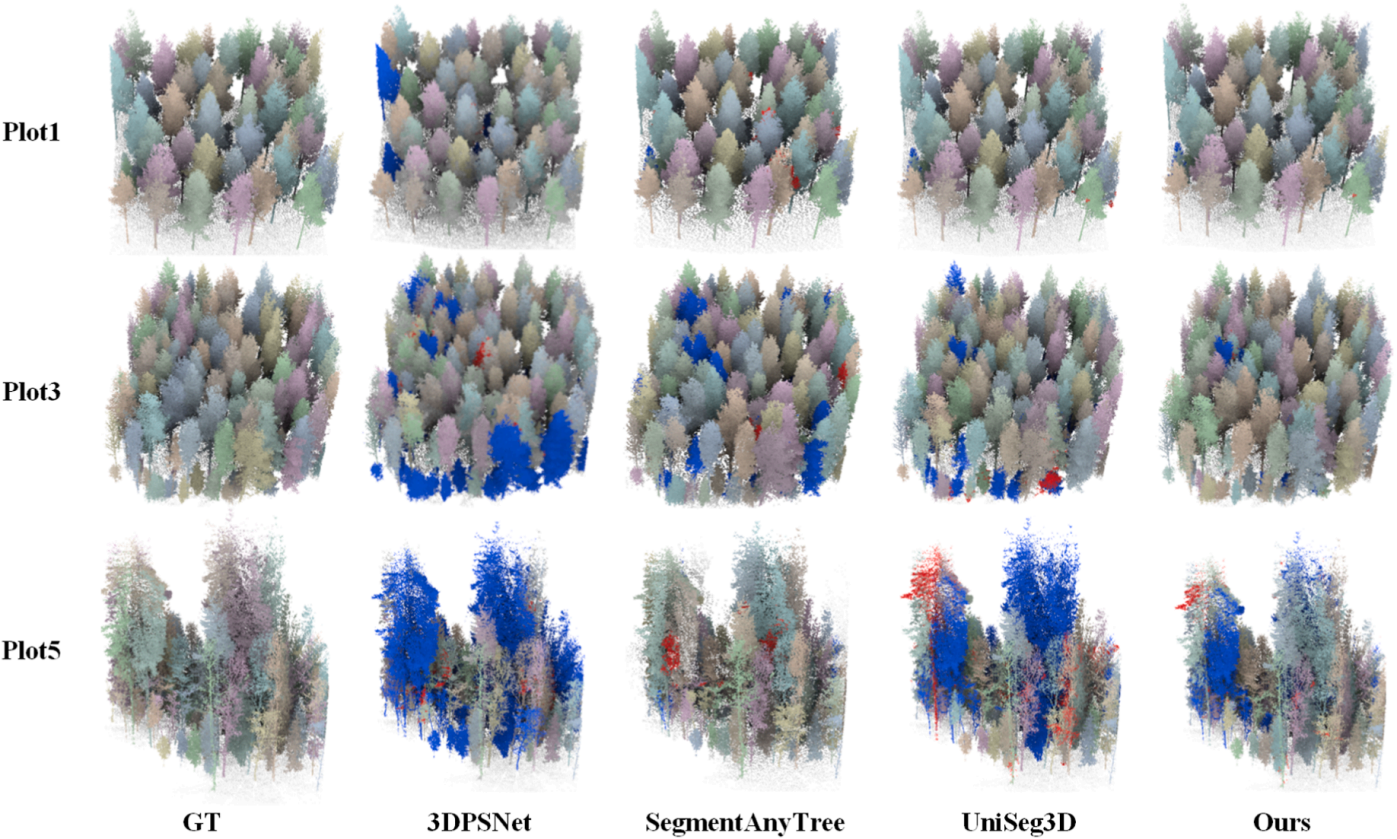
Qualitative comparison of different methods across three representative forest plots from the ForestSemantic dataset. Rows correspond to different forest plots, while columns show the GT and predictions from different methods. Red denotes false positives, and blue denotes false negatives with respect to the GT.

Fig. 2, Fig. 3 present qualitative comparisons between our method and representative baselines on FOR-instanceV2 and ForestSemantic, respectively, covering forest plots with distinct structural characteristics. Our method produces predictions that are visually more consistent with the GT, with sharper instance boundaries, higher structural completeness, and fewer false assignments.

On FOR-instanceV2, as shown in Fig. 2, the proposed method shows clear advantages in challenging scenes characterized by severe crown overlap, vertical stratification, and dense inter-tree contact. Compared with competing methods, our predictions exhibit more precise boundary delineation between neighboring trees, especially in canopy-overlap regions where other methods tend to generate fragmented instances or merge adjacent crowns.

On ForestSemantic, as shown in Fig. 3, similar trends can also be observed across plots with different stand structures. In relatively regular scenes such as Plot 1 and Plot 3, most methods can recover the dominant trees, but competing approaches still suffer from local leakage across adjacent crowns or incomplete instance predictions. In contrast, our method yields sharper instance boundaries and more spatially compact instance predictions, indicating that the proposed semantic-instance coupling strategy is effective in suppressing cross-instance confusion in crowded canopies.

A closer inspection suggests that the main advantage of our method lies in its stronger ability to preserve inter-instance boundaries while maintaining the overall integrity of individual trees. This property enables more reliable recovery of complete tree structures in cluttered stands. As a result, the proposed method better captures the spatial extent of target trees in most plots while reducing the tendency to absorb points from neighboring instances.

Nevertheless, Plot 5 in ForestSemantic remains challenging due to more complex vertical layering, heavier occlusion, and weaker local separation cues between adjacent trees. In this case, the advantage of our method becomes less pronounced, and several predicted instances still exhibit reduced completeness. This observation suggests that, although the proposed semantically guided framework is effective in improving instance separation in dense contact regions, further enhancement of global structural reasoning and occlusion-aware feature aggregation is still needed for highly complex multilayer forest stands. Representative local failure cases, including boundary confusion, partial omission under crown overlap, and sparse isolated misclassified clusters, are further analyzed in Supplementary Fig. S1.

### 3.4 Ablation studies

#### 3.4.1 Effectiveness of key components

Table 3 validates the effectiveness of the proposed key designs with SRM enabled. Both HSS and BCTD consistently improve individual tree segmentation performance, and their combination achieves the best overall results. Compared with the baseline, the full model improves Detection by 9.52 pp, reduces Omission by 9.52 pp, reduces Commission by 5.06 pp, improves F1-score by 7.71 pp, and improves mAP by 10.94 pp, showing that the proposed designs bring substantial gains in detection completeness, boundary discrimination, and overall instance quality.

**Table 3:** Ablation study on the key components of ForestSeg3D with SRM enabled. Best results are in **bold**, and second-best results are underlined.

HSS	BCTD	Individual Tree Segmentation (%)				
		Det *↑*	Omi *↓*	Com *↓*	F1 *↑*	mAP *↑*
Baseline		71.46	28.54	12.56	78.65	62.31
*✓*		79.21	20.79	10.81	83.91	71.87
	*✓*	76.68	23.32	12.40	81.78	69.27
DS [38]		79.29	20.71	12.45	83.22	70.47
	DML [39]	73.06	26.94	9.21	80.97	66.32
DS [38]	DML [39]	80.56	19.44	10.98	84.58	72.46
*✓*	DML [39]	76.85	23.15	8.33	83.61	70.97
***✓***	***✓***	**80.98**	**19.02**	**7.50**	**86.36**	**73.25**

HSS: Hierarchical Semantic Supervision; BCTD: Bidirectional Cross-Task Distillation; DS: Deep Supervision; DML: Deep Mutual Learning.

When used individually, HSS brings larger gains than BCTD on Detection, F1-score, and mAP, indicating that hierarchical semantic supervision is particularly effective for improving instance completeness and preserving tree structure. BCTD also improves the results consistently, and its contribution becomes more evident when combined with HSS. In particular, the combination of HSS and BCTD reduces Commission by 5.06 pp compared with the baseline and achieves the best value among all settings, suggesting that BCTD is especially effective in suppressing cross-instance confusion.

We further compare the proposed designs with two representative alternatives, i.e., deep supervision (DS) [38] and deep mutual learning (DML) [39]. Compared with DS, our HSS achieves a better overall trade-off, particularly in terms of Commission, F1-score, and mAP, verifying the advantage of the proposed coarse-to-fine semantic hierarchy over uniform multi-stage supervision. Compared with DML, the proposed BCTD achieves higher Detection and mAP, although its Commission error is higher, indicating a different trade-off between recall and precision. Although DS + DML achieves the closest performance among the alternatives, it still remains inferior to HSS + BCTD. Specifically, the full model further improves Detection by 0.42 pp, reduces Commission by 3.48 pp, improves F1-score by 1.78 pp, and improves mAP by 0.79 pp.

#### 3.4.2 Qualitative comparison of ablation results

**Fig. 4:**
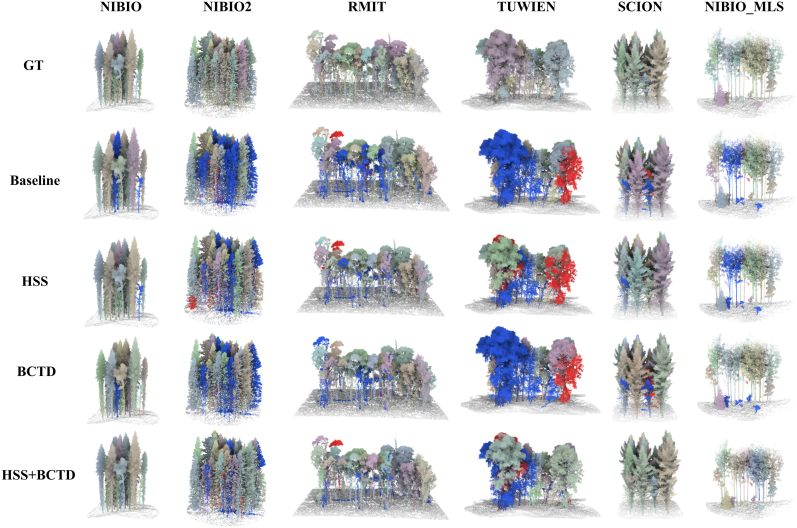
Region-level visual ablation across different forest regions. From top to bottom are the GT, baseline, HSS, BCTD, and HSS + BCTD, respectively. Red indicates false positives, and blue indicates false negatives relative to the GT.

**Fig. 5:**
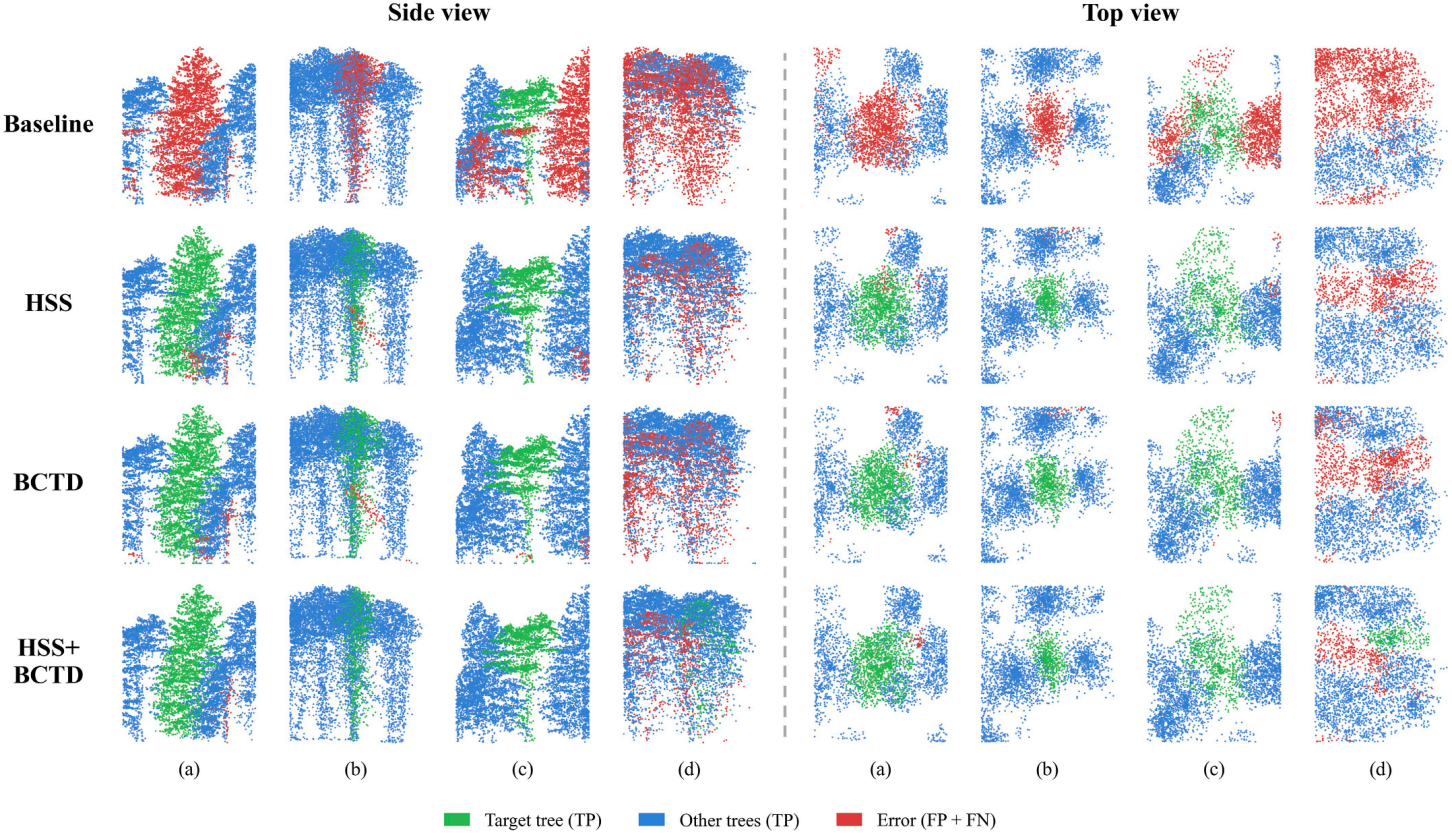
Paired side-view and top-view detail comparison for visual ablation. The left block shows side views and the right block shows the corresponding top views for the same four cases. Rows compare the baseline, HSS, BCTD, and HSS + BCTD. Green denotes correctly predicted target-tree points, blue denotes other correctly predicted tree points, and red denotes erroneous points, including false positives and false negatives.

Fig. 4 shows the region-level visual ablation results under different component settings, while Fig. 5 provides paired side-view and top-view detail comparisons for four representative cases. The baseline can capture many dominant trees, but it still exhibits typical failure modes, including incomplete target-tree delineation, cross-instance confusion in crowded canopy regions, and noisy predictions near boundaries or ground-adjacent regions. These errors are especially visible in the red points, which indicate false positives and false negatives.

At the region level, HSS mainly improves the structural completeness of individual trees. Compared with the baseline, predicted tree bodies become more continuous, and partially occluded trees are recovered more completely in several regions. BCTD, in contrast, mainly improves separation between neighboring trees by reducing semantic-instance conflicts near ambiguous boundaries. When HSS and BCTD are used together, the predictions show both improved structural completeness and cleaner inter-tree separation, yielding results closer to the GT.

The paired detail views in Fig. 5 further clarify the complementary roles of the two designs. In the side views, HSS more clearly recovers the vertical structure of target trees, especially in cases where the baseline misses lower or middle canopy components. This indicates that hierarchical semantic supervision helps preserve part-whole tree continuity. BCTD also improves the side-view results, but its effect is more evident in areas where adjacent trees are spatially close and prone to mask leakage.

In the corresponding top views, BCTD contributes more directly to crown-level boundary separation. It reduces horizontal overlap with neighboring trees and suppresses erroneous points around canopy contact zones, leading to cleaner target-tree regions. HSS also helps maintain more complete target crowns, but its main benefit is structural completeness rather than boundary refinement. HSS and BCTD address different but complementary failure modes: HSS enhances vertical and structural completeness, whereas BCTD strengthens boundary consistency. Their combination produces the most complete and spatially coherent predictions across both side and top views.

Additional sensitivity analyses of the hierarchical consistency weight *α*_3_ and the distillation scaling factor are provided in Supplementary Fig. S2, and the sensitivity of the overlap threshold *τ*_merge_ is reported in Supplementary Fig. S3.

#### 3.4.3 Ablation on scene-level region merging strategies

We further conduct an ablation to examine the effect of different scene-level region merging strategies. To isolate the influence of the scene-level merging rule, all variants in this ablation are evaluated under a controlled inference setting with the same crop-level predictions and fixed post-processing parameters, and differ only in the merging strategy. Therefore, the results in Table 4 are intended for within-table comparison among merging rules, rather than as a direct reproduction of the overall results reported in Table 1. Specifically, we compare three representative alternatives: an overlap-based merging strategy adapted from ForAINet [27], a score-based block merging strategy adapted from ForestFormer3D [30], and an NMS-based merging strategy inspired by OneFormer3D [36].

**Table 4:** Ablation on scene-level region merging strategies. All variants are evaluated under a controlled inference setting with the same crop-level predictions and fixed post-processing parameters, while only the merging rule differs. The results are intended for within-table comparison among merging strategies. Main values are in %, and values in parentheses denote relative differences to SRM in percentage points. Best results are in **bold**, and second-best results are underlined.

Strategy	Det *↑*	Omi *↓*	Com *↓*	F1 *↑*	mAP *↑*
Overlap	80.72 (−2.70)	19.28 (+2.70)	11.37 (+1.54)	84.49 (−2.17)	70.99 (−4.43)
Score	81.90 (−1.52)	18.10 (+1.52)	15.76 (+5.93)	83.06 (−3.60)	68.19 (−7.23)
NMS	82.66 (−0.76)	17.34 (+0.76)	15.64 (+5.81)	83.50 (−3.16)	68.01 (−7.41)
**SRM**	**83.42**	**16.58**	**9.83**	**86.66**	**75.42**

As shown in Table 4, SRM achieves the best overall performance among all merging strategies. Compared with the strongest alternative, i.e., the overlap-based strategy, SRM improves Detection by 2.70 pp, reduces Omission by 2.70 pp, reduces Commission by 1.54 pp, improves F1-score by 2.17 pp, and improves mAP by 4.43 pp. The score-based and NMS-based variants obtain relatively high Detection, but their larger Commission errors lead to lower F1-score and mAP.

These results indicate that the scene-level consolidation rule has a clear impact on segmentation quality, even when the crop-level predictions are fixed. Compared with alternatives that rely solely on geometric overlap, instance scores, or non-maximum suppression, SRM achieves a more favorable balance between detection completeness and false-positive control by jointly leveraging geometric overlap and instance-level semantic agreement during cross-region consolidation.

### 3.5 Structural parameter estimation

To examine whether improved instance segmentation translates into more reliable forest inventory outputs, we evaluate structural parameter estimation based on predicted tree instances. Following prior studies on LiDAR-based tree segmentation, we consider three representative attributes: tree height, crown diameter, and crown volume, which directly reflect the practical utility of segmentation for forest inventory applications [27,31]. For a fair comparison of structural parameter accuracy, we evaluate both methods on the common set of ground-truth trees correctly detected by both the baseline and ForestSeg3D. This isolates the effect of segmentation quality on attribute estimation, while detection completeness is evaluated separately by the instance segmentation metrics.

**Fig. 6:**
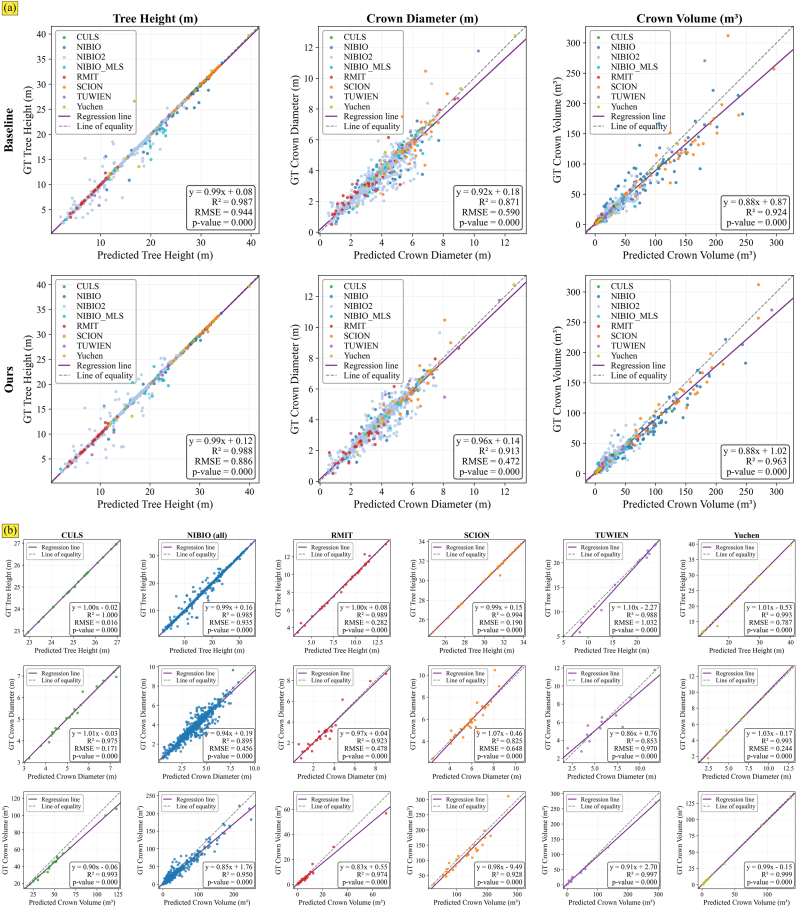
Structural parameter estimation results. (a) Overall comparison between the baseline and ForestSeg3D across all evaluated regions. Rows correspond to the baseline and ForestSeg3D, and columns correspond to tree height, crown diameter, and crown volume. (b) Region-wise results of ForestSeg3D, where columns represent forest regions and rows represent structural parameters. The purple line denotes the regression line, and the gray dashed line denotes the line of equality. In panel (b), NIBIO (all) combines the NIBIO-related regions.

Among these attributes, tree height is the most stable. As shown in Fig. 6(a), both the baseline and ForestSeg3D achieve near-perfect agreement with reference values for tree-height estimation, with regression slopes close to 1 and *R*^2^ values around 0.99. The performance difference is marginal (0.987 vs. 0.988 in *R*^2^, and 0.944 m vs. 0.886 m in RMSE), indicating that height estimation is largely insensitive to segmentation improvements. This is expected, as height estimation mainly depends on apex detection and ground reference rather than precise crown delineation.

In contrast, crown diameter is more sensitive to horizontal boundary quality. As shown in Fig. 6(a), ForestSeg3D improves *R*^2^ from 0.871 to 0.913, reduces RMSE from 0.590 m to 0.472 m, and increases the regression slope from 0.92 to 0.96, indicating reduced systematic underestimation of crown extent. This demonstrates that the proposed method achieves more accurate horizontal delineation, particularly in densely packed stands. A consistent trend is observed in the region-wise results in Fig. 6(b), where relatively regular regions (e.g., CULS and Yuchen) show near-ideal agreement, while structurally complex regions (e.g., NIBIO, RMIT, SCION, and TUWIEN) exhibit larger dispersion. This aligns with prior findings that crown-related measurements degrade under over- or under-segmentation in complex crowns [27].

Crown volume is the most sensitive attribute and provides the clearest evidence of downstream benefit. As shown in Fig. 6(a), ForestSeg3D improves *R*^2^ from 0.924 to 0.963 while maintaining a similar regression slope, indicating reduced underestimation compared with the baseline. The region-wise results in Fig. 6(b) show high accuracy in regular stands (e.g., CULS and Yuchen), but larger deviations in more complex regions, especially TUWIEN, where crowns are highly interleaved and vertically multilayered. This sensitivity is expected, as crown volume depends on the full 3D extent of the crown, and even small boundary deviations may lead to disproportionately large volumetric differences. In addition, crown volume inherently suffers from definition ambiguity, where slight variations in crown interpretation can lead to significant differences in estimated volume [27]. Unlike height or diameter, crown volume is strongly scale-dependent and more sensitive to the definition of crown extent. Large absolute differences may arise even when the predicted instance shape is visually reasonable. Therefore, we do not use RMSE as the primary criterion for crown-volume evaluation, and instead focus on regression slope, *R*^2^, and proximity to the 1:1 line when interpreting volume estimation results.

Together, Fig. 6 confirms that parameter estimation accuracy is closely tied to scene complexity, with crown-related attributes being substantially more sensitive to canopy overlap and vertical stratification than tree height. These results demonstrate that ForestSeg3D not only improves segmentation accuracy, but also enhances the reliability of downstream structural measurements, which is critical for practical forest inventory applications.

### 3.6 Limitations

The proposed HSS formulation follows a closed-world assumption consistent with the benchmark annotation protocol, where the fine-grained semantic classes are limited to Ground, Wood, and Leaf. Accordingly, the Non-Tree category in the coarse branch corresponds to Ground in the current experiments. During real-world inference on raw, unedited point clouds, additional objects such as anthropogenic structures or dense understory shrubs are not explicitly represented by the current semantic partition and may therefore be assigned to one of the existing classes. Handling such cases would require extending the annotation protocol to include additional Non-Tree categories during training or incorporating open-set or out-of-distribution detection strategies during inference.

## 4 Conclusion

In this study, we proposed ForestSeg3D, a semantically guided framework for individual tree segmentation from forest LiDAR point clouds. By integrating HSS and BCTD, ForestSeg3D improves both structural completeness and boundary discrimination in complex forest scenes: HSS provides coarse-to-fine semantic guidance for learning tree-aware structural representations, while BCTD enforces semantic-instance consistency to reduce cross-instance confusion and improve the separation of adjacent trees. For large-scale inference, SRM further consolidates overlapping predictions from independently processed sub-regions, improving scene-level instance consistency. Extensive experiments on the FOR-instanceV2 and ForestSemantic datasets demonstrate that ForestSeg3D achieves the best overall trade-off among existing methods in detection completeness, false-positive suppression, and F1-score, while maintaining competitive mAP. Ablation and qualitative analyses verify the complementary contributions of HSS and BCTD, and downstream evaluation further shows improved structural parameter estimation, particularly for crown-related attributes that are sensitive to instance quality. Nevertheless, extremely small, heavily occluded, and highly interleaved trees remain challenging, especially in multilayer forest scenes with sparse point density. Future work will focus on improving robustness under these difficult conditions and extending the framework to more diverse forest environments.

## Authors’ contributions

Ke Zhang: Writing – original draft, Visualization, Methodology, Conceptualization. Wenjun Zhang: Writing – review & editing, Software, Supervision. Weipeng Jing: Writing – review & editing, Funding acquisition, Resources, Supervision. Liang Li: Writing – review & editing, Project administration. Chao Li: Supervision, Writing – review & editing, Funding acquisition.

## Data availability

Some of the datasets, model weights, and code used in this study have been uploaded to the website: https://github.com/ikeke6/ForestSeg3D. The remaining datasets used in this study are publicly available from their original sources.

## Funding

This work was supported by the 10.13039/501100012166National Natural Science Foundation of China (32572045), the Key Research and Development Programme of Heilongjiang Province (2023ZX01A23), and the 10.13039/100006180Central Government Guided Local Science and Technology Development Fund under Grant (2024ZY0161).

## Declaration of competing interest

The authors declare that they have no known competing financial interests or personal relationships that could have appeared to influence the work reported in this paper.
